# Revisiting Waist Circumference: A Hypertension Risk Factor that Requires a More In-depth Understanding

**DOI:** 10.2174/011573403X290574240322041356

**Published:** 2024-03-27

**Authors:** Yue Su, Jin-yu Sun, Zhen-yang Su, Wei Sun

**Affiliations:** 1 Department of Cardiology, The First Affiliated Hospital of Nanjing Medical University, Nanjing, 210029, China

**Keywords:** Hypertension, waist circumference, body mass index, obesity, fat distribution, metabolism

## Abstract

As a major cause of various cardiovascular diseases, the prevalence of hypertension has been increasing in the past 30 years, leading to significant socioeconomic and health burdens. Obesity is one of the major risk factors for hypertension. Body mass index (BMI) is the most used anthropometric index to measure obesity in clinical practice and to assess the risk of obesity-related diseases. However, obesity is a heterogeneous disease, and the accumulation of fat in different body regions leads to differences in cardiovascular and metabolic risks. BMI only reflects the overall obesity but does not consider the distribution of fat and muscle mass. The limitation of BMI makes it insufficient to assess the risk of hypertension attributed to obesity. In addition, waist circumference is an easily obtainable anthropometric index to evaluate abdominal fat distribution. High waist circumference is an independent risk factor for various cardiovascular diseases and all-cause mortality regardless of BMI. Preliminary data indicate that waist circumference is significantly associated with the risk of hypertension at different BMI levels. However, routine measurement of waist circumference is currently not required in current clinical guidelines or is only recommended for obese populations, indicating an insufficient understanding of waist circumference. In this review, we summarize the measurement methods and diagnostic thresholds of waist circumference for abdominal obesity, the trend of central obesity prevalence, the superiority of waist circumference over other anthropometric indices, and recent cross-sectional and longitudinal studies on the association between obesity and hypertension.

## 1 INTRODUCTION

Hypertension is a cardiovascular syndrome primarily marked by elevated systemic arterial pressure, which stands as a major risk factor for various cardiovascular diseases. The 2017 American College of Cardiology/American Heart Association guidelines define hypertension as systolic blood pressure ≥ 130 mmHg and/or a diastolic blood pressure ≥ 80 mmHg [1], while most other guidelines use systolic blood pressure ≥ 140 mmHg and/or diastolic blood pressure ≥ 90 mmHg [2-4]. Over the past decade, the treatment and control of hypertension have improved substantially owing to the widespread availability of blood pressure measurement devices and antihypertensive medications. However, hypertension remains a significant health and economic burden due to the increasing number of patients, inadequate management of diagnosed individuals, low awareness rates, and high prevalence of masked hypertension [5, 6]. China has undergone rapid socioeconomic development and witnessed a substantial increase in life expectancy over the past 30 years. In tandem with these changes, the prevalence of hypertension has surged. It has climbed from 5.1% in 1958-1959 to 7.7% in 1979-1980, then to 11.3% in 1991, 18.8% in 2002, and 23.2% in 2012-2015 [7]. As hypertension continues its upward trajectory in China, numerous surveys and longitudinal studies have delved into its epidemiology, associated risk factors, and potential treatment strategies. These studies encompass national and regional investigations, such as the China Health and Nutrition Survey [8], the China Health and Retirement Longitudinal Study [9], and the Prospective Multi-omics Longitudinal Primary Hypertension Cohort [10].

Obesity, marked by excessive fat accumulation, has emerged as a major global health concern over recent decades [11-13]. An extensive analysis involving 128.9 million individuals spanning all age groups showcased a consistent rise in obesity rates worldwide from 1975 to 2016 [14]. According to the World Health Organization, the global obese population has nearly tripled since 1975. In 2016, over 1.9 billion adults (39%) were overweight, and more than 650 million adults (13%) were obese [15]. Research suggests that obesity is a significant contributor to hypertension, with increased body weight substantially elevating the risk of high blood pressure [16]. Particularly, central obesity, as compared to general obesity, is an obesity phenotype associated with higher metabolic risks, and its cardiovascular implications are becoming increasingly pronounced. Body mass index (BMI) is the most commonly used anthropometric measure of obesity in clinical practice. Previous guidelines recommended using BMI exclusively to assess the incidence of obesity-related diseases and mortality risk [17-20]. However, a growing number of studies suggest that BMI inadequately assesses or manages cardiovascular metabolic risks related to fat distribution. Based on the above, the expert consensus of the International Atherosclerosis Society and the International Society for Cardiometabolic Risk emphasizes that waist circumference should be routinely measured as a “vital sign” to comprehensively evaluate metabolic risks associated with fat distribution [21].

In this review, we summarize the methods for measuring waist circumference and the diagnostic thresholds for abdominal obesity. We also review the changing trends in the prevalence of abdominal obesity, compare the superiority of waist circumference over other anthropometric measures, and provide an overview of the cross-sectional and longitudinal studies on the association between obesity and hypertension in recent years.

## 2 MEASUREMENT OF WAIST CIRCUMFERENCE

**Fig. (1):**
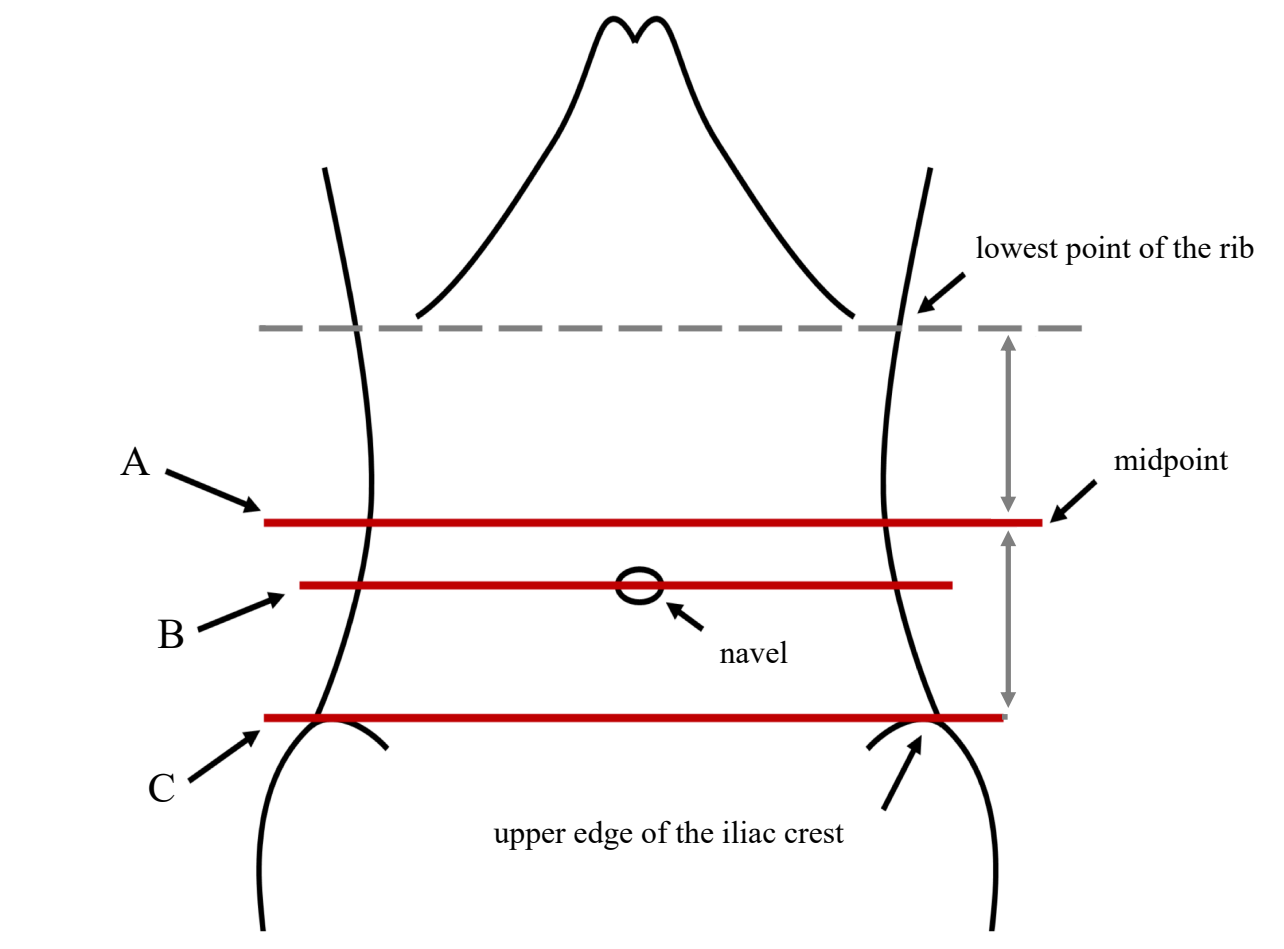
Commonly used waist circumference measurement positions. (**A)** Line: Level of the midpoint between the lowest point of the rib and the highest point of the iliac crest; (**B)** Line: Level of the navel; (**C**) Line: Level of the upper edge of the iliac crest.

Waist circumference is measured as follows: The subject stands upright, and an inelastic tape measure is horizontally wrapped around the waist, ensuring that the tape is snug against the skin but not compressing it. The waist circumference is measured at the end of exhalation. Presently, there is no consensus on the best method for measuring waist circumference. Different guidelines, expert consensus, and clinical studies slightly vary in where they measure waist circumference. Furthermore, there is no specific theoretical basis provided for the recommended measurement sites. Currently, three waist circumference measurement positions are the most widely adopted (Fig. **1**).

The World Health Organization recommends measuring at the level of the midpoint between the lowest point of the rib and the highest point of the iliac crest. The National Institutes of Health recommends measuring at the level of the upper edge of the iliac crest. The level of the navel is also a commonly used measurement point in clinical research [22, 23].

Various methods of waist circumference measurement yield differing results. When measuring at the midpoint between the lowest rib and the highest point of the iliac crest, as well as at the level of the upper edge of the iliac crest, there are no significant differences in waist circumference values among males. However, among females, a variance of approximately 1.8 to 2.0 cm has been observed [24, 25]. In a study involving 542 participants, Mason *et al.* [25] measured waist circumference at both the midpoint level of the lowest point of the rib and the highest point of the iliac crest and at the level of the upper edge of the iliac crest, respectively. These measurements were then used to assess the prevalence of abdominal obesity, with values above 102 cm for men and 88 cm for women considered indicative of abdominal obesity. In males, both measurement methods resulted in a 32% prevalence of abdominal obesity. In contrast, among females, the prevalence was 47% when measured at the level of the upper edge of the iliac crest and 41% when measured at the level of the midpoint level [25]. These studies suggest that different methods of waist circumference measurement have a limited impact on male waist circumference values but significantly affect female waist circumference values.

The impact of varying waist circumference measurement methods on research outcomes has been a subject of ongoing concern among researchers. A meta-analysis of 120 clinical studies investigated the impact of various waist circumference measurement methods on the correlation with metabolic disease prevalence and mortality. The results showed that the method of measuring waist circumference did not significantly influence the research outcomes in any of the subgroups across sample size, gender, age, and ethnicity [21]. This indicates that when using waist circumference to assess the risk of metabolic-related diseases, the chosen measurement approach had a limited impact on the study results. Furthermore, there is currently no established theoretical basis for the selection among the three commonly employed measurement levels. Consequently, when evaluating metabolic risk in relation to waist circumference, researchers may not need to consider the influence of different measurement methods on research conclusions.

## 3 CLASSIFICATION CRITERIA FOR ABDOMINAL OBESITY DEFINED BY WAIST CIRCUMFERENCE

Lean *et al.* [26], in a cross-sectional study involving 2,206 Caucasian individuals, found that a waist circumference of ≥ 102 cm for males or ≥ 88 cm for females, effectively identified individuals with a BMI ≥ 30 kg/m^2^ and a waist-to-hip ratio ≥ 0.95 (males) or ≥ 0.80 (females), with a sensitivity of 96% and a specificity of 98%. These waist circumference thresholds of ≥ 102 cm for males and ≥ 88 cm for females were initially adopted by the National Institutes of Health in the United States to diagnose abdominal obesity. The race-specific waist circumference-based diagnostic criteria for abdominal obesity are shown in Table **1** [27]. While variations in the association between waist circumference and health risks have been observed across different BMI categories [28], the current obesity risk classification system recommends using a consistent waist circumference threshold across all BMI categories.

However, these diagnostic criteria were initially designed to use waist circumference as an alternative to BMI for identifying obesity without considering the clinical significance of waist circumference [29]. Ardern *et al.* [30] constructed abdominal obesity waist circumference diagnostic criteria for identifying individuals at high risk of coronary events in different BMI categories based on data from 18,254 individuals from the United States and Canada. The Ardern waist circumference criteria are as follows: BMI 18.5-24.9 kg/m^2^, waist circumference ≥ 90 cm (males)/≥ 80 cm (females), BMI 25-29.9 kg/m^2^, waist circumference ≥ 100 cm (males)/≥ 90 cm (females), BMI 30-34.9 kg/m^2^, waist circumference ≥ 110 cm (males)/≥ 105 cm (females), and BMI ≥ 35 kg/m^2^ and waist circumference ≥ 125 cm (males)/≥ 115 cm (females). In subsequent study, Bajaj *et al.* [31, 32] compared Ardern waist circumference values with conventional measurements for the assessment of all-cause mortality in 5,453 individuals with a high cardiovascular metabolic risk. The results indicated that the Ardern waist circumference values significantly outperformed traditional thresholds in predicting all-cause mortality [31]. However, this threshold was designed to assess coronary risk, and its predictive value for hypertension and other metabolic diseases remains unclear. Therefore, there is still a need for large-scale prospective data from diverse ethnic groups to support updates to diagnostic thresholds for abdominal obesity, enabling a more effective realization of the clinical value of waist circumference in relation to fat distribution-related metabolic risk.

## 4 TRENDS IN THE PREVALENCE OF ABDOMINAL OBESITY

The ongoing monitoring of obesity prevalence is essential for refining obesity management strategies and evaluating metabolic risk within populations. Numerous studies have reported obesity trends in the United States and various other nations [32-34]. Nevertheless, clinical practice seldom includes routine waist circumference measurements, resulting in limited reporting on the prevalence of abdominal obesity based on this metric.

Based on data from the National Health and Nutrition Examination Survey (NHANES) in the United States, our team investigated the dynamic changes in abdominal obesity among the American population over the past two decades [35]. Our research showed a notable, time-dependent increase in the prevalence of abdominal obesity from 2001-2002 to 2017-2018 in the United States. Specifically, the age-adjusted prevalence of abdominal obesity surged from 57.58% to 67.33% in females and from 39.07% to 49.73% in males [35]. Furthermore, we observed significant variations in waist circumference among individuals with similar BMI values [35]. Li *et al.* [36] observed substantial changes in waist circumference and abdominal obesity prevalence in the mainland Chinese population. The average waist circumference swelled from 80.7 cm in 2007 to 83.5 cm in 2017, with a mean change of 3.4 cm (95% confidence interval: 0.6-6.2 cm, *p* = 0.02). Concurrently, the prevalence of abdominal obesity prevalence escalated significantly from 25.9% in 2007 to 35.4% in 2017 (*p* = 0.0002). However, there was no significant alteration in the proportion of individuals with a slight increase in BMI (BMI, 23-25 kg/m^2^) between 2007 and 2017 (20.3% *vs*. 20.8%, *p* = 0.05). Ian Janssen *et al.* [37] conducted an observational study in Canada from 1981 to 2007 and showed that for individuals with a BMI of 25 kg/m^2^, waist circumference increased by 1.1 cm in men and 4.9 cm in women. Research spanning various time periods in China (1993-2011), the United Kingdom (1992-2008), and Mexico (1999-2012) has indicated that waist circumference exhibits more pronounced growth compared to BMI [38]. Significant increments in waist circumference, surpassing changes in BMI, were observed in sub-analyses across diverse age groups, genders, and ethnicities [39]. These findings emphasize the importance of incorporating waist circumference measurements alongside BMI since the prevalence of abdominal obesity continues to rise. Given these trends, integrating waist circumference into global obesity surveillance is crucial for a more comprehensive assessment of obesity-related health risks.

## 5 THE ADVANTAGE OF WAIST CIRCUMFERENCE IN ASSESSING OBESITY-RELATED METABOLIC RISK

There are numerous anthropometric indicators used to assess obesity, such as BMI, waist-to-hip ratio (WHR), waist-to-height ratio (WHtR), and waist circumference. An increasing body of research suggests that waist circumference possesses distinct advantages compared to other anthropometric measures in evaluating obesity-related metabolic risk.

Currently, BMI stands as the most widely utilized metric for gauging obesity and assessing associated cardiovascular risks. BMI was introduced in the 1830s by the Belgian statistician Adolphe Quetelet. It is calculated by dividing the body weight of an individual in kilograms by the square of height in meters, expressed as BMI = body weight/height^2^. A large number of studies have confirmed that overall obesity with a high BMI is an independent risk factor for a multitude of diseases, including cardiovascular diseases, diabetes, and cancer [9, 40-42]. However, recent large-scale population cohort studies have brought to light the existence of the “obesity paradox”, indicating a J-shaped or U-shaped curve relationship between BMI and the risk of various diseases [43-46]. In a meta-analysis involving 250,152 coronary artery disease patients, it was found that individuals with slightly higher BMI had lower risks of cardiovascular and all-cause mortality when compared to those with normal or obese BMI [45]. Obesity is a heterogeneous disease, and variations in fat distribution across different body regions lead to disparities in cardiovascular metabolic risk [47, 48]. Excessive abdominal fat accumulation represents a high-risk metabolic phenotype, contributing to varying susceptibilities to hypertension among obese individuals [49]. This paradox may arise from the limitations of BMI in assessing fat distribution. BMI cannot differentiate between fat and muscle weight, offering only a crude measure of overall obesity. Consequently, relying solely on BMI-based obesity standards is inadequate when evaluating and managing individual metabolic risks associated with obesity.

Kissebah *et al.* [50] classified the fat distribution into upper and lower body fat accumulation, using WHR for assessment. High or low WHR values, respectively, indicate upper or lower body fat accumulation. Epidemiological studies have revealed a close association between high WHR values and increased risks of various metabolic diseases and mortality [51-53]. However, changes in both the numerator (waist circumference) and the denominator (hip circumference) impact WHR. As emphasized by Allison *et al.* [54], utilizing ratios can significantly impact the interpretation and validity of statistical results. Similarly, waist-to-height ratio (WHtR) has also been proposed as a screening tool for obesity-related metabolic risk. This measure takes height into account as a negative correlation, but whether the effect of height on metabolic risk is independent of the underlying mediators remains unclear. Additionally, height displays only a weak correlation with waist circumference (men: correlation coefficient 0.051, *p* = 0.018; women: Correlation coefficient 0.036, *p* = 0.063). Consequently, when assessing metabolic risk using waist circumference, the influence of height can be disregarded [55]. In fact, height is associated with genetic and environmental exposure early in life; thus, WHtR is more widely used to assess metabolic risk in children or adolescents [55]. Both WHR and WHtR are based on WC, emphasizing the importance of the latter. When employing WHR or WHtR to monitor shifts in regional fat distribution, accurately discerning the nature of these changes becomes challenging. This limitation restricts its clinical applicability [21].

Waist circumference stands as a simple method for assessing abdominal fat distribution, recognized for its ease of standardization and clinical application. Waist circumference exhibits a stronger correlation with the absolute amount of intra-abdominal or visceral fat compared with BMI, WHtR or WHR, rendering it a more comprehensive tool for evaluating metabolic risks associated with abdominal fat distribution [29]. Many studies have shown that waist circumference is an independent risk factor for various metabolism-related diseases and mortality [28, 56-62]. These include cardiovascular diseases (such as hypertension, atherosclerosis [63], heart failure), stroke, type 2 diabetes [64], dyslipidemia (abnormal blood lipid levels, fatty liver [65]), female infertility [61], and metabolic syndrome. A prospective cohort study of 46,651 Europeans aged 24 to 99 years examined the association between different measures of obesity and CVD mortality. Results showed that during a mean follow-up of 7.9 years, there were 3435 deaths, of which 1409 were due to cardiovascular disease [58]. A systematic review and meta-analysis involving over 680,000 European participants revealed that waist circumference above 95 cm in men and 80 cm in women was associated with all-cause mortality [66]. Notably, even within the normal BMI range (20.0~24.9 kg/m^2^), surpassing these waist circumference thresholds was linked to a higher relative risk of all-cause mortality [66]. Cerhan *et al.* [28] conducted a pooled analysis of 11 prospective cohort studies encompassing 650,386 individuals aged 20 to 83 years from the United States, Australia, and Sweden. After a median follow-up of 9 years, 78,268 participants had deceased. Following adjustments for age, BMI, study, smoking status, alcohol consumption, and physical activity, a positive correlation emerged between waist circumference and all-cause mortality. Among males, each 5-centimeter increase in waist circumference corresponded to a 7% increase in mortality risk, while in females, every 5-centimeter increase in waist circumference translated to a 9% rise in mortality risk. Notably, within various BMI subgroups, an increase in waist circumference significantly increased the risk of all-cause mortality [28]. These results consistently demonstrate the association between abdominal obesity and increased risk of cardiovascular mortality and all-cause mortality.

Interestingly, some studies have shown that when BMI and waist circumference are both considered in a single regression model, waist circumference remains an independent risk factor for cardiovascular disease, while BMI becomes a protective or neutral factor [28, 46, 56, 67-69]. Coutinho *et al.* [46] carried out a prospective study of an average follow-up time of 2.3 years involving 14,284 adults with cardiovascular disease. After measuring waist circumference, gender, age, smoking, diabetes, and hypertension, BMI exhibited a negative correlation with the mortality risk (HR 0.64, 95% credible interval 0.59-0.69). However, after adjusting for BMI, gender, age, smoking, diabetes, and hypertension, the mortality risk in the high-waisted group was obviously increased compared with that of the low-waisted group (HR 1.29, 95% credible interval 1.20 to 1.39). In addition, when waist circumference and BMI were both included in the analysis, the health risks associated with waist circumference became even more significant [56, 70]. Pischon *et al.* [56] carried out a large prospective research with 359,387 participants and found the highest waist circumference quintile (≥ 102.7 cm for males and ≥ 89.0 cm for females) is strongly related to all-cause mortality in models not adjusted for BMI (RR value 1.33; 95% confidence interval: 1.24-1.44). Moreover, after correcting for BMI, the relevance between high waist circumference and all-cause mortality becomes even more pronounced (RR value 2.05; 95% confidence interval: 1.80-2.33). Consequently, some researchers suggest that when analyzing waist circumference and obesity-related metabolic risk, it is necessary to adjust for the influence of BMI to thoroughly assess the relevance between waist circumference and disease incidence and mortality [28, 57, 70]. The application of both BMI and waist circumference may allow for a more comprehensive assessment and management of obesity-related adverse health events [71-77]. Klein *et al.* [72] proposed that the change in the relationship between waist circumference (WC) and health outcomes was less pronounced compared to that of BMI and health outcomes, but BMI is more influenced by demographics. Likewise, Rao *et al*. [73] contend that BMI categorization lacks precision, with cutoff values diverging significantly from actual clinical conditions and inadequately capturing the full spectrum of cardiovascular disease risk. Regardless of whether adjusting WC by BMI or BMI by WC, the value of analyzing both indicators together is evident in coronary heart disease [74] and hypertension [75]. Wang *et al.* [77] creatively proposed the waist-to-BMI ratio (WtBR) as an anthropometric measure to better assess obesity-related risk. In summary, the combination of BMI and waist circumference can identify the highest-risk phenotype of obesity, far surpassing the utility of measuring either indicator alone. BMI serves as a comprehensive measure reflecting overall body fat distribution, while WC better represents abdominal or visceral obesity.

Although the two cannot be algebraically computed, the difference between them lies in subcutaneous fat and muscle content. Therefore, for a given waist circumference, a higher BMI confers protective health effects through increased subcutaneous fat tissue in the lower body, which also effectively explains the “obesity paradox [21]”. The classification of populations by Yang *et al.* [76] into four subgroups-comorbid obesity, abdominal obesity alone, general obesity alone, and non-obesity-based on cutoff values of BMI and WC aligns with our perspective seamlessly. In the case of abdominal obesity at normal BMI (low BMI and high waist circumference), for example, this particular metabolic phenotype implies low muscle mass, low subcutaneous fat distribution in the extremities, the presence of excess accumulation of visceral and ectopic fat, and poor cardiorespiratory fitness. Individuals with this profile face an elevated risk of cardiovascular disease [78, 79].

The above suggests that waist circumference can provide a more effective assessment of obesity-related metabolic risk compared to WHR and BMI and is an important complement to BMI. Besides, some abdominal obesity indices, such as Chinese visceral adiposity index (CVAI) [80], weight-adjusted-waist index (WWI) [81], and bioelectrical impedance [82], may have better predictive ability for hypertension and cardiometabolic risk than waist circumference. However, these indicators are all limited to a specific population of hypertensive patients and have advantages in a specific metabolic disease, while they are rarely mentioned in other populations or metabolic problems.

## 6 INVESTIGATIONS ON THE CORRELATION BETWEEN OBESITY AND HYPERTENSION

Forepassed research on the relationship between obesity and hypertension predominantly relied on BMI, often overlooking the distribution of visceral or abdominal cavity fat. Limited clinical studies have delved into the connection between waist circumference and hypertension, frequently categorizing waist circumference for examining the cross-sectional link between abdominal obesity and hypertension [59, 83-85]. Recently, a growing number of studies have attempted to analyze waist circumference as a continuous variable to explore the longitudinal correlation between waist circumference and the prevalence of hypertension using data from prospective studies.

Sun *et al.* [59] analyzed data from a cross-sectional study comprising 27,894 individuals from the U.S. population to explore the correlation between waist circumference and hypertension. This study used the diagnostic threshold for hypertension of 130/80 mmHg recommended by the American College of Cardiology. After correcting for covariates, such as BMI, gender and age, a significant positive correlation was observed between waist circumference and hypertension, with an OR of 1.28 (95% confidence interval, 1.18-1.40) for individuals under 45 years and 1.23 (95% confidence interval, 1.15-1.33) for those over 45 years. Restricted cubic spline plots further validated the dose-response relationship between waist circumference and hypertension, with the morbidity of hypertension increasing with increasing waist circumference [59]. In a subsequent study, a subgroup analysis of 8,795 individuals with normal BMI (18.5-24.9 kg/m^2^) was conducted to investigate the influence of waist circumference on developing hypertension. When waist circumference was analyzed as a continuous variable, the OR for it (per 10 cm) was 1.27 (95% confidence interval, 1.12-1.44); when waist circumference was analyzed as a four-categorical variable, the OR for the latter 25% of the waist circumference group (Q4 group) was 3.87 (95% confidence interval, 1.59-10.34) compared to the first 25% of the waist circumference group (Q1 group) [60]. Notably, the follow-up research established that even in individuals with normal BMI and lipid levels, waist circumference remained significantly associated with hypertension [79]. Similarly, Ren *et al.* [86] examined 10,719 adult participants from the cross-sectional data of the China Health and Nutrition Survey to research the correlation between abdominal obesity and hypertension among those with normal weight. In individuals with normal BMI, the abdominal obese group had a higher risk of hypertension than the normal waist circumference group (OR 1.49, 95% confidence interval 1.14-1.95) [86]. Kuciene R *et al*. [87] examined the associations between waist circumference and high blood pressure among Lithuanian adolescents aged 12-15 years. Compared with the first quartile, the adjusted odds ratios in the highest quartiles of waist circumference were 6.44 in boys and 3.54 in girls. These cross-sectional studies initially showed the correlation between waist circumference and hypertension. However, demonstrating a causal relationship between hypertension and abdominal obesity is challenging with cross-sectional designs. Therefore, we conducted longitudinal research in subsequent work to further investigate the connection between high baseline waist circumference and the new-onset hypertension incidence.

Sun *et al.* [9] analyzed data from the China Health and Retirement Longitudinal Study to explore the relationship between WC and the development of hypertension. The findings indicate that elevated WC serves as a risk factor for the onset of hypertension. This discovery holds significant implications for hypertension prevention and management, particularly in a country like China, where hypertension prevalence is notable. After adjusting for BMI, gender, age, low-density lipoprotein levels, stroke, diabetes, heart disease, use of glucose-lowering medications, alcohol consumption and smoking status, each 10-centimeter increase in waist circumference corresponded to an 18% higher risk of developing hypertension. In subgroup analyses of different BMI levels, age, and gender, there was a significant association between waist circumference and the prevalence of hypertension [9]. However, the above research was conducted among individuals aged 45 years and older, with a mean of 57.4 Further validation is required to generalize these findings to the general population. Zhao *et al.* [88] carried out a cohort research in a rural area of Luoyang, including 10,265 individuals without hypertension at baseline. After 6-year follow-up, there were 2,027 cases of hypertension. Participants were divided into 4 groups based on changes in waist circumference, which were ≤ -2.5%, -2.5% to 2.5%, 2.5% to 5% and > 5%. Compared with the control group (-2.5% to 2.5%), the relative risk of hypertension was 1.34 (95% confidence interval, 1.15 to 1.57) for males with a > 5% increase in waist circumference, and in females, it was 1.28 (95% confidence interval, 1.10 to 1.50) [88]. Wang *et al.* [89] conducted a study that revealed, after adjusting for BMI, a 10-centimeter increase in waist circumference was related to a 0.98 mmHg increase in systolic blood pressure (in both men and women) and a 1.13 mmHg increase in diastolic blood pressure (in men) and a 0.74 mmHg increase (in women). A longitudinal study followed 6,096 Chinese individuals with normal blood pressure for 12 years [90]. Between 1997 and 2009, the abdominal obesity prevalence rose from 17.3% to 39.4%, and 26.8% of participants developed hypertension. After correcting for BMI and other covariates, logistic regression analysis indicated that the risk of hypertension in the abdominal obesity group was 79% (95% confidence interval, 36% to 135%) higher compared to the non-abdominal obesity group [90].

## CONCLUSION

A large body of research has shown the importance of waist circumference in assessing the risk of developing hypertension. Nevertheless, its routine measurement is not yet a standard practice in current clinical protocols, or it is merely recommended as an additional assessment for individuals with obesity. The current depth of research exploring the correlation between waist circumference and metabolic diseases is insufficient, and the clinical utilization of waist circumference remains inadequate. To establish the relationship between waist circumference and hypertension at varying BMI levels and to devise the most effective hypertension risk stratification method, further longitudinal data are imperative. Such research will provide high-quality evidence from evidence-based medicine to support the routine use of waist circumference measurements in hypertensive patients.

## Figures and Tables

**Table 1:** Race-specific diagnostic criteria for abdominal obesity waist circumference (adapted from Alberti *et al.* [27]).

**Race**	**Waist Circumference Threshold (Males)**	**Waist Circumference Threshold (Females)**
American	≥ 102 cm	≥ 88 cm
Chinese	≥ 90 cm	≥ 85 cm
Canadian	≥ 102 cm	≥ 88 cm
Japanese	≥ 85 cm	≥ 90 cm
Middle easterner	≥ 94 cm	≥ 80 cm
Central American/South American	≥ 90 cm	≥ 80 cm

## LIST OF ABBREVIATIONS

BMI: Body Mass Index
NHANES: National Health and Nutrition Examination Survey
WHtR: Waist-to-Height Ratio
WHR: Waist-to-Hip Ratio
